# Restoring fertility in premature ovarian insufficiency: Targeting the ovarian stroma with antifibrotic drugs

**DOI:** 10.1002/ctm2.70799

**Published:** 2026-10-23

**Authors:** Yu Zhao, Kui Liu

**Affiliations:** ^1^ Department of Cardiology, Ningbo Medical Centre Lihuili Hospital Ningbo University Ningbo Zhejiang China; ^2^ Laboratory of Food Nutrition and Clinical Research Institute of Seafood Zhejiang Gongshang University Hangzhou China; ^3^ Department of Obstetrics and Gynecology Li Ka Shing Faculty of Medicine The University of Hong Kong Hong Kong SAR China

Premature ovarian insufficiency (POI) has long been recognized as a significant clinical challenge affecting women, with profound implications for reproductive health, quality of life and long‐term health outcomes. Recent epidemiological data showed that the prevalence of POI is substantially higher than previously estimated, ranging from 3.5% to 3.7%,^1^ notably higher than the earlier estimate of 1%.^2^ When combined with early menopause, the collective prevalence reaches 12.2%, reflecting a considerable public health burden.^1^ Despite these alarming numbers, therapeutic options for POI‐associated infertility remain extremely limited. Although substantial progress has been made in understanding the molecular mechanisms that govern primordial follicle activation,^3^, ^4^ which have enabled the development of in vitro activation (IVA) and led to successful pregnancies and live births.^5^, ^6^ To date, no oral medication has yet been approved to stimulate the growth of small follicles in POI patients. This underscores the pressing demand for a safe, non‐invasive alternative.

## 1 FINERENONE AS A SAFE AND EFFECTIVE ORAL OPTION FOR RESTORING FERTILITY

Drug repurposing offers a pathway to rapidly translate existing safe drugs into new clinical applications.^7^ To this end, Lin and colleagues established a systematic four‐step screening and repurposing pipeline to identify clinically viable candidates. They first screened a Food and Drug Administration (FDA)‐approved drug library of 1297 compounds and identified 10 candidate drugs that upregulate Kit ligand (*Kitl*) expression in ovarian cells.^8^ Upregulation of *Kitl* in pre‐granulosa cells is a key event that initiates follicle activation,^4^ suggesting these drugs have the potential to activate follicular growth. Using neonatal mouse ovarian culture and kidney capsule transplantation models, Lin et al. showed that finerenone, an oral drug approved for chronic kidney disease, effectively promotes primordial follicle activation and development. In aged mice, both *intra‐ovarian* injection and oral gavage of finerenone increased secondary follicle counts. Oral finerenone also increased cumulative offspring production in 6‐month‐old mice over the 18‐week observation period, with no detectable adverse effects on oocyte maturation, fertilization, embryonic development, or offspring health.^8^ These safety data support the potential clinical use of finerenone for fertility restoration in POI patients.

## 2 A PARADIGM SHIFT FROM FOLLICLES TO STROMAL FIBROSIS

Lin et al. recognized that the ovarian microenvironment, especially the stromal compartment, plays a critical but underappreciated role in regulating follicular development.^8^ The ovarian stroma contains general components like immune cells, blood vessels, nerves and lymphatics, along with ovary‐specific elements such as surface epithelium, tunica albuginea, rete ovarii, hilar cells and various fibroblast‐like and interstitial cells.^9^ This stromal network not only provides structural support but also delivers biomechanical and signalling cues that regulate follicle activation and growth.

Ovarian tissue fibrosis and impaired follicular development are the core pathological features of POI.^10^ Lin et al. demonstrated that finerenone reduces stromal‐to‐granulosa cell communication by downregulating collagen‐mediated signalling in ovarian stromal cells. A critical experiment using lipid nanoparticle (LNP)‐encapsulated siRNA to knock down the collagen gene *Col1a1* showed that reducing collagen deposition alone was sufficient to promote follicle activation.^8^ This suggests that stromal collagen acts as a negative regulator of follicle activation, and that fibrosis‐related collagen deposition imposes a physical and signalling barrier to follicular development. Relieving this fibrotic constraint represents a key mechanism by which finerenone promotes follicle growth (Figure 1). This hypothesis marks a shift from directly targeting follicles to modifying the restrictive stromal environment that surrounds them.

**FIGURE 1:**
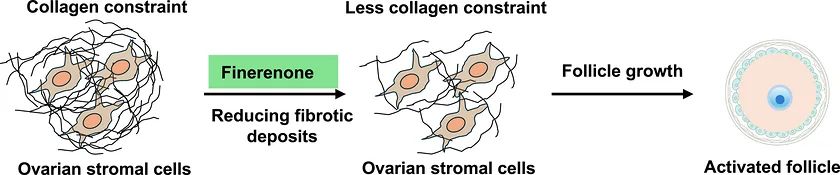
Schematic illustration of finerenone‐mediated follicle activation by reducing stromal collagen constraints. Excessive collagen deposited by stromal cells imposes physical and signalling constraints that suppress follicle activation and development (left). Finerenone reduces this fibrotic constraint by downregulating collagen expression in stromal cells (middle), thereby creating a permissive microenvironment that allows follicle growth (right).

Notably, finerenone is not the only agent with this effect. Several other FDA‐approved oral antifibrotic drugs, including nintedanib, obeticholic acid, ruxolitinib, pacritinib and fedratinib, also enhanced follicle activation, supporting a class effect.^8^ The efficacy of multiple antifibrotic agents with distinct mechanisms further suggests that antifibrotic activity is the key driver of follicle activation.

## 3 CLINICAL EVIDENCE AND IMPLICATIONS

This approach was then tested in a pilot clinical study of 14 patients with POI‐associated infertility. Patients received oral finerenone at 20 mg twice weekly lower than the 20 mg/day regimen used for chronic kidney disease, with follow‐up of 3 to 7 months. Strikingly, follicular development waves were observed in all 14 patients, with a total of 80 antral follicle‐like structures observed across 72 ultrasound monitoring sessions. Oocyte retrieval was performed in 17 cycles, yielding 12 oocytes from 8 patients. The retrieval rate (70.59%) and oocyte maturity rate (75.00%) were comparable to conventional IVF outcomes in the general population. Among five married patients who proceeded to fertilization, three cleavage‐stage embryos were successfully obtained.^8^ These encouraging findings need validation in larger, multicentre, randomized controlled trials to confirm the efficacy, optimal dosing and long‐term safety of finerenone and other antifibrotic agents for POI.

Traditional approaches to treating infertility in POI have focused almost exclusively on the follicles themselves, typically attempting to activate dormant follicles or stimulate follicular growth through hormonal manipulation. While these strategies have yielded some success in other contexts but have fallen short in POI, because the underlying problem extends beyond the follicle. These findings provide the first clinical evidence that an oral antifibrotic drug finerenone can restore follicular development and enable oocyte retrieval in POI patients. This study also establishes ovarian fibrosis as a druggable target, expanding treatment options for POI, since these agents already have well‐characterized safety and tolerability profiles.

## 4 CONCLUSION

This study provides preclinical and clinical evidence that relieving ovarian fibrosis is an effective strategy for fertility restoration in POI, validating ovarian fibrosis as a promising therapeutic target. The findings indicate that existing safe drugs could be repurposed for POI treatment, potentially offering an accessible therapeutic option. This research offers new hope for POI patients and points to a new direction in reproductive medicine.

## AUTHOR CONTRIBUTIONS

Yu Zhao drafted the Letter and was responsible for organizing the manuscript content. Kui Liu critically reviewed the manuscript during the preparation of the final version. Both authors read and approved the final version of the manuscript.

## CONFLICT OF INTEREST STATEMENT

The authors declare no conflict of interest.

## ETHICS STATEMENT

This Invited Letter does not report new primary research involving human participants or animals. Therefore, ethical approval and informed consent were not required. Any clinical data mentioned are derived from the previously published study by Lin et al. and are cited accordingly.
